# Impact of environmental factors on the population dynamics, density and foraging activities of *Odontotermes lokanandi* and *Microtermes obesi* in Islamabad

**DOI:** 10.1186/2193-1801-2-349

**Published:** 2013-07-29

**Authors:** Abdul Sattar, Muhammad Naeem, Ehsan ul-Haq

**Affiliations:** Department of Entomology, Pir Mehr Ali Shah Arid Agriculture University Rawalpindi, Rawalpindi, Pakistan; Insectary-Biological Control; labs, National Agriculture Research Center, Islamabad, Pakistan

**Keywords:** *O. lokanandi*, *M. obesi*, NIFA TERMAPs, Temperature, Relative humidity, Rainfall

## Abstract

Affect of different environmental factors i.e., temperature, relative humidity and precipitation on population dynamics, density and foraging activities of *Microtermes obesi* Holmgren and *Odontotermes lokanandi* Chatarjee and Thakur (Isoptera: Termitidae) were studied from March 2010 to July 2012 in Islamabad. A total of 1200 poplar wooden stakes was used for monitoring the termite activities in Islamabad. The results showed that 65 out of 1200 poplar wooden stakes were found infested by both species i.e. *M. obesi* and *O. lokanandi.* Both species were interacting with each other in the experimental field and *O. lokanandi* was found significantly dominant. Mean yield per trap ranged from 0.83 ± 0.20 gm to 1.12 ± 0.28 gm and 0.35 ± 0.09 gm to 0.82 ± 0.19 gm for *M. obesi* and *O. lokanandi* in the field, respectively. *M. obesi* and *O. lokanandi* in 1.0 gm sample ranged from 539.83 ± 2.21 to 567.83 ± 9.41 and 407.67 ± 4.75 to 424.5 ± 1.15 individuals, respectively. Population of workers ranged from 93.53 ± 1.73 to 97.68 ± 0.40 and 91.69 ± 1.42 to 98.41 ± 0.50 percent for *M. obesi* and *O. lokanandi*, respectively.

Positive and significant correlation was found among atmospheric temperature, precipitation and both subterranean termite species i.e., *M. obesi* and *O. lokanandi*; however, the correlation was found non significant and negative between relative humidity and foraging activities of both termite species.

Moreover, correlation was found positive and significant between atmospheric temperature and percent workers of *M. obesi*; while negative and non-significant between atmospheric temperature and percent workers of *O. lokanandi*. Negative and significant correlation was noted between relative humidity and percent workers of *M. obesi*; whereas, positive and significant correlation was recorded between relative humidity and percent workers of *O. lokanandi*. Positive and non-significant correlation was recorded between precipitation and percent workers of *M. obesi*; while positive and significant correlation was observed between precipitation and percent workers of *O. lokanand*.

## Introduction

Termites or white ants are eusocial roaches (Inward *et al.*^2007^), belonging to the order Blattodea. They are polymorphic living in colonies that comprise of reproductive, soldiers and workers. The queen is very much bigger than the king, is capable of laying eggs at the rate of 36,000 a day for as long as 50 years. Worker termites perform taking care of the brood, maintaining and repairing the nest, and foraging for food (Krishna 1969), moreover, they feed other caste i.e., soldiers and functional reproductive (Grassé Grasse 1939; Noirot and Noirot-Timothee 1969). Termites are a large group of organisms of which there are greater than 2600 species (Kambhampati and Eggleton 2000). Subterranean termites live in large colonies and can range from about 0.2- 5 million individuals (Grace *et al*^1989^) and the colony grows slowly for many years (Bignell and Eggleton 1998).

Subterranean termites cause significant building and urban structural damages throughout the world, especially in the tropical and sub-tropical regions (1969; Edwards and Mill 1986; Pearce 1997), they damage forestry and wide range of agriculture crops including cash crops such as maize, wheat, groundnuts, and rice; and pastures (Dawes-Gromadzki 2005).

Different trapping techniques have been described to aggregate and collect subterranean termites. Su and Scheffrahn (1986) described an underground collection unit consisting of a wooden box within a short length of polyvinylchloride (PVC) pipe, with a plastic cap, that is buried below the soil surface at urban environment to monitor subterranean termites. Esenther (1980) buried corrugated fiberboard to collect *R. flavipes*, and La Fage *et al.*(1983) reported a technique of extracting subterranean termites from infested wood by placing a short length of PVC containing a roll of moistened corrugated fiberboard on top of the wood. Many scientists have used excavated nest to collect data, although this procedure excludes termites in peripheral foraging galleries (Holdaway *et al.*^1935^; Gay and Greaves 1940; Rohrmann 1977; Ohiaqu 1979; Collins 1981; Howard *et al.*^1982^). Terminologists used ground stakes to monitor termite foraging activities (Esenther and Beal 1974; 1978; Su *et al*. 1982; Jones 1989).

Atmospheric temperature and rainfall have been found correlated with seasonal foraging activities of termites (Abensperg-Traun 1991; Haagsma and Rust 1995; Rust *et al.*^1996^; Dibog et al. 1998; Haverty *et al.*^1999^; Evans and Gleason 2001; Daves-Gromadski and Spain 2003; Mesenger and Su 2005; Moura *et al.*^2006^). Foraging activities of *Coptotermes lacteus* (Froggat) was found correlated with both soil and air temperature (Evans and Gleason 2001). Studies have shown that seasonal changes in the foraging behavior of subterranean termites may influence the efficacy of baiting programs due to decline of activities during winter (Ripa *et al.*^2007^; Haverty *et al*^2010^).

The objective of the present study was to determine whether changes in temperature, relative humidity and precipitation affect the population dynamics, density and foraging activities of *O. lokanadi* and *M. obesi* in Islamabad.

## Materials and methods

Ecological study of subterranean termites was conducted in Islamabad; the Federal Capital of Pakistan. Geographically, it is situated at northern latitudes 33° 42’ 0” and eastern longitudes 72° 10’ 0” lying at an altitudes of 457 to 610 m above sea level. Its elevation is 507 meters (1,663 feet). Islamabad lies in the sub-tropical, sub-humid continental climatic zone. Total area of the Federal Capital of Pakistan is 906 square Km and is bounded on the west by Attock, Hazara in the north, Rawalpindi in the south and poonch of Azad Kashmir in the east. The climate is characterized by hot summers and cold winters, with some frost events in January. The mean maximum temperature in the hottest month of June is 40°C; while the mean minimum temperature of January is 3°C. The mean annual rain fall is about 1000 mm, 70 percent of which falls during the summer monsoon season (July, August and September) and remaining 30 percent falls in winter (December, January and February). The soil is slightly alkaline, non-saline, loamy in texture, low in organic matter and major nutrients with exception of available K (Nizami *et al.*^2004^). The plant community of Islamabad consists of *Justicia adhatoda* L.*, Mangifera indica* L.*, Tamarix aphylla* (L.) H. Karst.*, Acacia modesta* Wall.*, Dodonaea viscose* (L.) Jacq.*, Zizyphus nummularia* (Burm. F.) Wight & Arn.*, Pinus roxburghii* Sarg.*, Apluda mutica* L.*, Quercus incana* Bartr.*, Woodfordia fruticosa* (L.) Kurz.*, Broussonetia papyrifera* (L.) Venten.*, Fiscus palmata* Forsk. *and Dicliptera roxburghiana* Nees (1987).

### Survey

**Figure 1:**
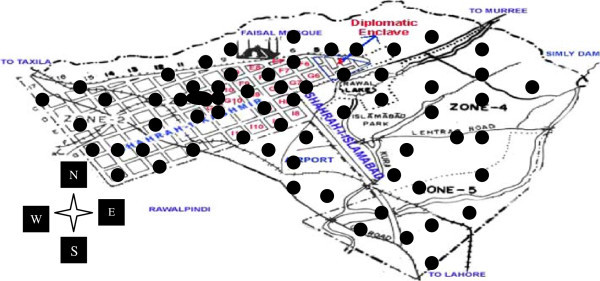
**Location map of the study areas.** The black dots show NIFA-TERMAPS.

, followed by the procedure used by Su and Scheffrahn 1988. A total of 1200 monitoring stakes was driven into the soil of infested areas of Islamabad and these stakes were checked fortnightly. Out of the 1200 stakes placed in the ground, typically only 65 were infested by two termite species i.e., *O. lokanandi* and *M. obesi* and these infested stakes were replaced with “NIFA-TERMAPs” (Figure 1).

### Population dynamics of subterranean termites

Stakes (2.5 × 4 × 28 cm) (thickness width length) of poplar wood were buried in termite infested areas of Islamabad, and were checked fortnightly. When any stake was found infested by termite, a “ NIFA-TERMAP” which, consist of a PVC pipe (8 mm thickness × 15 cm dia × 20 cm length) buried in the soil having a bundle of 5 poplar wooden slices (1.3 × 8 × 15 cm) wrapped in blotting paper covered with earthen lid (Salihah *et al.,*^1993^) was installed on that point. The wooden stakes as well as “NIFA-TERMAPs” were checked fortnightly and the infested traps by termites were replaced with a new one. The infested traps were brought to the laboratory to separate the termites from the soil and debris, collected termites were weighed. The number of soldiers and workers were also determined in one gram termite sample. The total numbers of termites were obtained by multiplying the number counted in one gram with the total weight. From each trap sample of 5–10 workers and soldiers were preserved in 80% alcohol for identification of the species. Identification of termites from each and every trap were done on each episode by using the key of Chaudhry *et al.* (1972).

### Ecology of foraging termites

Foraging ecology was studied by counting the number of termites captured by termite trap named “ NIFA-TERMAP” (Salihah *et al.*^1993^) under the prevailing temperature, relative humidity and rain fall of the experimental site. Air temperature and relative humidity were measured with the help of Hygrotherm and the data of rainfall was collected from Meteorological Department of Islamabad. The effect of relative humidity, temperature and rainfall were also studied on the caste composition of foraging group of termites.

Statistical computation was performed by using Co-Stat and MStat-C. Means were separated by using Duncan’s Multiple Range Test.

## Results and discussion

### Population dynamics of subterranean termite

Tables 1 and 2 shows that mean ± SE yield of *Microtermes obesi* and *Odontotermes lokanandi* varied among each trap, i.e., it ranged from 0.83 ± 0.20 gm to 1.12 ± 0.28 gm and 0.35 ± 0.09 gm to 0.82 ± 0.19 gm, respectively. Our results indicate that such variation exists in foraging sites of different as well as a single colony. There seems to be three factors: i. termites did not like the high moisture content of the soil; ii. the distance from the colony that worker would travel; iii. the termite soldiers apparently do not distribute homogeneously within their gallery system. Lower yield of termites was found in traps, which were installed in wet or irrigated field or away from the colony. While, higher yield of termites was recorded in traps, which were installed in dry field or near to the colony. Similarly, the mean number of individuals in 1.0 gm sample varied greatly in case of both species. It ranged from 539.83 ± 2.21 to 567.83 ± 9.41 and 407.67 ± 4.75 to 424.5 ± 1.15 individuals per sample for *M. obesi* and *O. lokanandi*, respectively (Tables 1 and 2). This variation is due to the size and age of the individuals of foraging groups. The traps which were installed near the colony were found to have adult workers, soldiers as well as nymphs and therefore a large number of individuals were recorded in 1.0 gm sample. The traps which were installed faraway from the colony were found to have the adult workers, soldiers and therefore, less number was recorded in 1.0 gm sample. Variations were also found in mean number of individuals of *M. obesi* per gram sample of the different traps. A considerable intra-specific variation exists in termite colonies (Su and Fage 1984). Feeding at baits was negative correlated with soil moisture for *Coptotermes getroi* (Wasmann) and positive correlated with soil moisture for *Heterotermes longiceps* (Synder) (Santos *et al.*^2010^).

**Table 1:** **Mean yield (g), mean number of termites in 1.0 gm sample and mean percent workers in foraging group of*M. obesi*collected from “NIFA TERMAPs” installed in urban environment (Islamabad) from September 2010 to September 2012**

Trap No.	Termite		Worker proportion (%)	Trap No.	Termite		Worker proportion (%)
	Wt (g)	Number			Wt (g)	Number	
10	1.01 ± 0.23	553.67 ±8.81	97.20 ± 0.57	418	0.96 ± 0.19	547.00 ± 8.71	95.90 ± 1.16
14	1.05 ± 0.24	548.50 ± 7.57	97.68 ± 0.40	641	0.85 ± 0.21	563.67 ± 11.76	96.36 ± 1.14
15	0.91 ± 0.23	542.83 ± 8.12	97.51 ± 0.58	720	1.03 ± 0.24	549.50 ± 5.26	95.25 ± 0.92
26	1.12 ± 0.28	567.83 ± 9.41	97.06 ± 0.69	741	0.88 ± 0.22	542.83 ± 7.64	95.28 ± 1.42
70	0.83 ± 0.20	560.67 ± 10.15	96.52 ± 1.21	756	0.93 ± 0.22	554.17 ± 6.64	95.58 ± 1.98
75	0.96 ± 0.23	547.83 ± 8.84	96.02 ± 0.63	757	0.91 ± 0.22	549.00 ± 8.16	95.99 ± 1.10
79	0.86 ± 0.21	557.00 ± 11.99	96.41 ± 1.11	811	1.00 ± 0.23	540.17 ±2.39	94.79 ± 1.15
170	1.05 ± 0.25	555.50 ± 7.42	94.40 ± 1.19	812	0.93 ± 0.22	560.67 ± 8.49	95.33 ± 1.31
210	0.89 ± 0.21	551.50 ± 7.90	96.53 ± 1.11	822	0.93 ± 0.21	546.33 ± 5.94	95.39 ± 1.54
211	0.94 ± 0.23	554.00 ± 6.57	96.48 ± 1.00	825	0.88 ± 0.21	539.83 ± 2.21	96.22 ± 1.61
255	1.02 ± 0.26	549.00 ± 7.19	95.06 ± 1.33	833	0.93 ± 0.21	557.67 ±6.45	94.01 ± 1.55
333	1.02 ± 0.26	549.67 ± 7.98	93.53 ± 1.73	838	0.92 ±0.24	547.67 ± 4.57	96.67 ± 1.02
334	0.90 ± 0.22	541.83 ± 5.68	93.95 ± 1.36	844	1.06 ± 0.25	547.17 ±6.57	96.82 ± 1.14
335	0.90 ±0.22	550.33 ± 7.87	96.07 ± 1.72	845	1.04 ± 0.25	542.00 ± 8.05	96.99 ± 0.74
410	0.97 ± 0.23	546.50 ± 5.30	96.07 ± 1.28

**Table 2:** **Mean yield (g) and mean number of termites in 1.0 gm sample and mean percent workers in foraging group of*O. lokanandi*collected from “NIFA TERMAPs” installed in urban environment (Islamabad) from September 2010 to September 2012**

Trap No.	Termite		Worker proportion (%)	Trap No.	Termite		Worker proportion (%)
	Wt (g)	Number			Wt (g)	Number	
3	0.62 ± 0.13	418.17 ± 3.09	94.88 ± 1.09	415	0.68 ± 0.14	412.00 ± 2.67	96.08 ± 1.15
7	0.64 ± 0.14	422.00 ± 2.28	96.76 ± 1.11	417	0.76 ± 0.16	414.00 ± 2.25	97.15 ± 1.02
10	0.43 ± 0.10	410.33 ± 1.63	95.57 ± 1.37	418	0.43 ± 0.10	416.17 ±2.02	96.19 ± 1.23
11	0.70 ± 0.16	413.50 ± 3.56	96.66 ± 1.11	523	0.71 ± 0.17	419.50 ± 3.36	97.29 ± 0.72
13	0.78 ± 0.17	422.83 ± 3.18	95.90 ±1.12	524	0.63 ± 0.15	421.50 ±3.49	96.76 ± 1.16
17	0.66 ± 0.15	416.67 ± 4.33	95.15 ± 1.41	724	0.79 ± 0.17	418.17 ± 4.42	96.23 ± 1.16
18	0.77 ±0.16	419.83 ± 4.95	96.61 ± 1.08	728	0.65 ± 0.14	416.83 ±2.63	93.73 ± 1.56
25	0.68 ± 0.15	416.17 ± 4.74	95.56 ± 1.32	730	0.71 ± 0.14	417.17 ±2.56	93.56 ± 1.64
26	0.53 ± 0.15	415.83 ± 2.26	95.24 ± 1.09	731	0.66 ± 0.15	419.50 ± 3.31	94.44 ± 1.13
51	0.68 ±0.15	422.67 ±3.13	96.15 ± 1.12	732	0.73 ± 0.15	416.17 ± 4.94	96.06 ± 1.82
54	0.75 ± 0.15	418.33 ± 2.47	94.67 ± 1.71	756	0.38 ±0.09	416.67 ± 2.23	98.34 ±0.40
73	0.66 ±0.14	416.67 ± 3.85	96.01 ± 1.32	791	0.68 ±0.15	420.17 ± 2.46	98.23 ± 0.59
75	0.43 ± 0.11	412.50 ± 2.24	91.69 ± 1.42	792	0.64 ± 0.14	418.00 ± 4.67	97.39 ± 1.23
115	0.71 ± 0.15	414.17 ± 2.50	95.97 ± 1.15	798	0.69 ± 0.15	412.00 ± 2.11	96.77 ± 1.63
117	0.70 ± 0.16	417.17 ± 4.40	97.61 ± 0.82	810	0.68 ±0.15	414.33 ± 1.89	97.56 ± 0.71
118	0.69 ± 0.15	412.17 ± 3.87	97.10 ± 1.32	811	0.45 ± 0.10	417.50 ± 3.02	95.33 ± 1.71
119	0.63 ± 0.15	418.50 ± 3.10	96.86 ± 1.46	826	0.56 ±0.13	420.33 ± 2.55	95.25 ± 1.85
170	0.39 ± 0.09	413.17 ± 2.97	96.56 ± 1.14	828	0.71 ± 0.14	415.67 ± 2.67	94.16 ± 1.62
213	0.73 ± 0.16	424.50 ± 1.15	98.18 ± 0.55	829	0.66 ± 0.14	418.33 ± 3.40	98.39 ± 0.77
256	0.82 ± 0.19	417.17 ± 2.58	97.36 ± 1.09	841	0.67 ±0.15	410.00 ± 3.10	96.76 ± 0.54
258	0.62 ± 0.15	417.00 ± 4.48	98.18 ± 1.24	844	0.35 ± 0.09	407.67 ± 4.75	96.09 ± 1.62
332	0.59 ± 0.13	423.67 ± 3.17	96.28 ± 0.72	845	0.41 ± 0.09	417.83 ± 3.32	96.09 ± 0.73
333	0.44 ± 0.10	417.83 ± 3.20	98.41 ± 0.50	847	0.65 ± 0.15	418.33 ± 3.42	96.40 ± 1.09

Comparison on the number of individuals of the two species per sample shows a great variation. A significantly greater number of *M. obesi* was observed as compared to *O. lokanandi*. The minimum number (539.83 ± 2.21) of the former species is more than the mean maximum number (424.5 ± 1.15) of the latter (Tables 1 and 2). This variation is due to the different size of the two species. Individuals of *M. obesi* are smaller in size than individuals of *O. lokanandi* so, more individuals were counted in 1 gm sample. The two termite species were also found different greatly in yield per trap and number per 1.0 gm sample. The maximum yield of *M. obesi* per trap was 1.12 ± 0.28 gm, while of *O. lokanandi* 0.82 ± 0.19 gm. This variation shows that the termite population in the colony of *M. obesi* is high as compare to *O. lokanandi* so, more termites come to the foraging point. The number of individuals in a termite colony varies with species (Badawi *et al.*^1984^).

### Foraging ecology of subterranean termites

**Figure 2:**
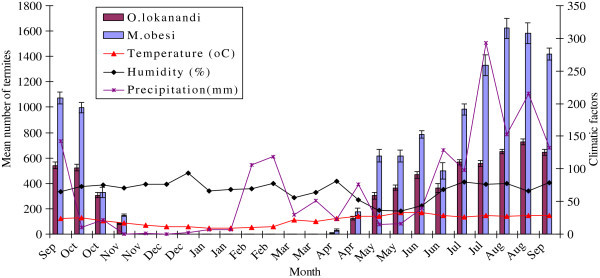
**Effect of atmospheric temperature, relative humidity and precipitation on Mean ± SE number of*M. obesi*and*O. lokanandi*collected through “NIFA TERMAPs” installed in Islamabad during September, 2010 to September, 2012.**

Foraging activity of *M. obesi* and *O. lokanandi* colonies appeared to be dependent on temperature. On average, greater percentage of the wooden stakes were attacked in summer than in winter. Analysis of the number of termites captures by NIFA-TERMAPs and environmental factors indicated that maximum temperature and precipitation influenced the foraging activity of either species of termites, while relative humidity did not influence the foraging activity (Figure 2). No biomass of both termite species was collected in winter months when the temperature was low (December, January, February and March), while the relative humidity was recorded high. Moreover, much water was retained in the soil during the winter period of the study. When the temperature increased, maximum numbers of termite were captured (Figure 2). Subterranean termites will not forage in areas where soil surface temperature is too hot or too cold (Haverty *et al*. 1974; La Fage et al. 1976; Smith and Rust 1994).

The result shows positive and significantly different correlation among atmospheric temperature, precipitation and both subterranean termite species (*M. obesi* and *O. lokanandi*), however, correlation was recorded negative and non-significantly different among relative humidity and both termites species i.e. *M. obesi* and *O. lokanandi* (Table 3).

**Table 3:** **Correlation between environmental factors and population of termites species captured through “NIFA TERMAPs” from urban environment (Islamabad)**

Termites species	Atmospheric temp (°C)	Relative humidity (%)	Precipitation (mm)
*M. obesi*	r =0.717*, P = 0.00	r = −0.030 ns, P = 0.889	r = 0.608*, P = 0.002
*O. lokanandi*	r = 0.766*, P = 0.00	r = − 0.077 ns, P = 0.721	r = 0.557*, P = 0.004

* = Significantly different at 5% level of significance: *ns* Non-significant.

In the present studies peaked foraging activities of subterranean termite were recorded in summer months when the temperature and precipitation were high, ground and atmospheric temperature is favorable for termites foraging in summer and fall. Rainfall during the evaluation period was also contributed to the termites being more active. Rain makes soil moist, and termites need moisture to survive and develop. The correlation of termite catch with climatic conditions indicated that the activity of *O. obesus, O. horni* and *O. feae* was significantly correlated with minimum temperature, maximum soil temperature, minimum relative humidity, total rainfall and number of rainy days (Shanbhang and Sundararaj 2011). Foraging activities of termites have been correlated with both temperature and rainfall (Evans and Gleason 2001). Johnson and Whitford (1975) and Ueckert et al. (1976) have however, reported that foraging activity is correlated to considerable extent with soil moisture and temperature. Abushaman and Al-Houty (1988) have also reported positive correlation between termite activity and soil moisture content. Potter (2004) stated that subterranean termites are very vulnerable to desiccation and require a constant supply of moisture. In addition, temperature has strong influence on termite foraging and seasonal activities. Lenz and Evans (2002) stated that subterranean habits are widely assumed to reduce adverse effect of weather.

### Caste composition of foraging groups of subterranean termites

The results revealed that the foragers captured throughout the observation period were predominantly workers. Mean population of workers ranged from 93.53 ± 1.73 to 97.68 ± 0.40 and 91.69 ± 1.42 to 98.41 ± 0.50 percent for *M. obesi* and *O. lokanandi*, respectively (Table 1 and Table 2). The variation in percent workers suggests that environmental factors viz., temperature; relative humidity and rainfall affect the ratio of the workers to soldiers. The results (Table 4) shows positive and significant correlation between atmospheric temperature and percent workers of *M. obesi*; while negative and non-significant between atmospheric temperature and percent workers of *O. lokanandi*. Negative and significant correlation was noted between relative humidity and percent workers of *M. obesi*; whereas, positive and significant correlation was recorded between relative humidity and percent workers of *O. lokanandi*. Positive and non-significant correlation was recorded between precipitation and percent workers of *M. obesi*; while positive and significant correlation was observed between precipitation and percent workers of *O. lokanand*. *Coptotermes getroi* (Wasmann) was found negative correlated with soil moisture; whereas *Heterotermes longiceps* (Synder) was noted positive correlated with soil moisture (Santos *et al.*^2010^).

**Table 4:** **Correlation between environmental factors (atmospheric temperature**, **relative humidity and precipitation) and caste composition of termites**

Termites species	Atmospheric temp (°C)	Relative humidity (%)	Precipitation (mm)
*M. obesi*	r =0.184*, P = 0.005	r = −0.208*, P = 0.001	r = 0.069 ns, P = 0.292
*O. lokanandi*	r = −0.090 ns, P = 0.084	r = 0.174*, P = 0.001	r = 0.159*, P = 0.002

* = Significantly different at 5% level of significance. *ns* Non-significant.

The caste composition in social insects can be influenced by environmental factors such as temperature. (Henderson 1998; Mao *et al.*^2005^; Scharf *et al.*^2007^). Furthermore, caste composition in termite colony or foraging groups of termites are known to vary with time of day, season, species, and colony size or age (Bodot 1970; Sands 1965; Bouillon 1964).

In the present studies more workers were collected as compared to soldiers in each observation from both species. This shows that the worker termites come to forage in large number as compared to soldiers. Nutting (1970) recorded 4% soldiers and 96% non soldiers in a foraging group of *H. aureus*. Foraging group of *Gnathamitermes perplexus* contain mainly workers and only about 0.4% soldiers (Nutting *et al.*^1973^).
